# The Hydrolysis of Carbonyl Sulfide at Low Temperature: A Review

**DOI:** 10.1155/2013/739501

**Published:** 2013-07-15

**Authors:** Shunzheng Zhao, Honghong Yi, Xiaolong Tang, Shanxue Jiang, Fengyu Gao, Bowen Zhang, Yanran Zuo, Zhixiang Wang

**Affiliations:** Department of Environmental Engineering, College of Civil and Environmental Engineering, University of Science and Technology Beijing, Beijing 100083, China

## Abstract

Catalytic hydrolysis technology of carbonyl sulfide (COS) at low temperature was reviewed, including the development of catalysts, reaction kinetics, and reaction mechanism of COS hydrolysis. It was indicated that the catalysts are mainly involved metal oxide and activated carbon. The active ingredients which can load on COS hydrolysis catalyst include alkali metal, alkaline earth metal, transition metal oxides, rare earth metal oxides, mixed metal oxides, and nanometal oxides. The catalytic hydrolysis of COS is a first-order reaction with respect to carbonyl sulfide, while the reaction order of water changes as the reaction conditions change. The controlling steps are also different because the reaction conditions such as concentration of carbonyl sulfide, reaction temperature, water-air ratio, and reaction atmosphere are different. The hydrolysis of carbonyl sulfide is base-catalyzed reaction, and the force of the base site has an important effect on the hydrolysis of carbonyl sulfide.

## 1. Introduction

With the rapid development of ecomy, energy supply and demand have become increasingly prominent; the scientific use of high-sulfur energy received extensive attention. Among the chemical raw material gases, which are made from coal, oil and natural gas, sulfur compounds can generally be divided into two major categories of organic sulfur and inorganic sulfur. Organic sulfur contains carbonyl sulfide (COS), carbon disulfide (CS_2_), thiophene, mercaptan, and so forth [1]. Inorganic sulfur is mainly hydrogen sulfide (H_2_S). COS, accounted for approximately 80% to 90% of the total organic sulfur, is normally regarded as a significant poison which can cause the deactivation of the industrial catalyst. Even only a trace amount of COS can result in the deactivation of catalysts and can lead to corrosion of reaction equipments [2–4]. For instance, as little as 4 mg of sulfur per gram of catalyst on the surface of the Fe-Cu-K catalyst decreases the activity by ca. 50% in the Fischer-Tropsch process [5]. Furthermore, not only does COS cause economic loss, but also affects the environment. COS is thought to be the most abundant sulfur-containing gas in the troposphere. Apart from volcanic injection, tropospheric COS is the main source of sulfur in the stratosphere leading to the stratospheric sulfate layer. The reactions of H, OH, and O (3P) with COS are known as important chemical sinks for consumption of this compound [6]. It has been proved to be a major source of acid rain when oxidized to sulfur oxide and to promote photochemical reactions [7–9]. In addition, in the past decades, much attention was drawn to the sources and sinks of carbonyl sulfide (COS) in the atmosphere due to the important role of COS in the formation of stratospheric sulfate aerosols [10–15]. Therefore, the removal of carbonyl sulfide is the main problem to solve in the process of feed gas deep purification.

Currently, the principal methods of carbonyl sulfide removal include hydrogenation, hydrolysis, absorption, adsorption, photolysis, and oxidation, [16–22]. Hydrolysis method, which has the advantages of low reaction temperature, no consumption of the hydrogen source, and few side effects, attracts a lot of researches at home and abroad. Carbonyl sulfide hydrolysis research mainly includes two aspects: (1) research and development of hydrolysis catalyst; (2) research on reaction kinetics and mechanism. The two complement each other and promote each other. The preparation of the catalyst is to use a suitable carrier load on a certain amount of an active ingredient. At present, the carriers of COS hydrolysis catalysts mainly contain two categories: (1) metallic oxides, including a composite metal oxide such as *γ*-Al_2_O_3_, TiO_2_ and manganese iron composite metal oxides; (2) nonmetallic oxides, mainly refer to activated carbon. Active ingredients which can load on the carrier include alkali metal, alkaline earth metal, transition metal oxides, rare earth metal oxides, mixed metal oxide, and nanoscale metal oxides. Some catalysts with relatively superior performance have been researched and developed at home and abroad so far.

The researches on reaction kinetics and mechanism not only can accelerate the development of hydrolysis catalyst with good performance and high activity, but also can provide a theoretical basis for the reactor design. Consequently, domestic and foreign researchers researched and developed catalyst. They also studied the carbonyl sulfide hydrolysis reaction kinetics and mechanism and made a number of research achievements.

In this paper, the development of catalyst for carbonyl sulfide catalytic hydrolysis, reaction kinetics, and reaction mechanism has been summarized systematically.

## 2. Catalysts for COS Hydrolysis

### 2.1. Catalyst Carrier

Great catalyst carrier should have proper specific surface area, good heat resistance, and mechanical strength. At the moment, COS hydrolysis catalyst carrier basically has two kinds. One is a delegate with Al_2_O_3_ and TiO_2_ metal oxides. Another kind of non-metallic oxide is a delegate with activated carbon. In addition, with the development of material science, the researchers also have developed many new types of catalyst carrier, such as cordierite.

#### 2.1.1. Metal Oxide

Metallic oxide carrier of COS hydrolysis catalyst refers mainly to Al_2_O_3_ and TiO_2_. In addition, Fe-Mn metal oxides have obtained the good effect in COS removal. Among them, the Al_2_O_3_ as the present study more in-depth and widely used catalyst has some characteristics of large specific surface area, high surface activity, good thermal stability, and so forth. Al_2_O_3_ itself has a certain activity for the hydrolysis of COS. The patent CN1069673 introduces a kind of Al_2_O_3_ catalyst in which COS hydrolysis conversion rate would be 51.2% under the condition of not adding any additives [28, 29]. However, this kind of catalyst sulphate resistance is poorer. We increase surface alkali center number and intensity by dipping a certain amount of alkaline components on the surface to that further improve the COS hydrolysis activity and to improve the service life and the catalytic properties such as resistance to poisoning [30]. Al_2_O_3_ itself is same as TiO_2_ to have COS hydrolysis function, and its sulfate resistance is better than Al_2_O_3_.

The catalyst with TiO_2_ as the carrier has a great activity, and high mechanical strength at low temperature [31]. Although the specific surface area of TiO_2_ is low, the price is high; it is not an easy molding industry, and the industrial applications are chief based on A1_2_O_3_ catalyst. However, by a small amount of TiO_2_ modulation, the A1_2_O_3_ catalyst ability greatly enhanced resistance to sulfur poisoning. Composite carrier was found that it is of granular distribution and good dispersion by scanning electron microscope.

In recent years, the mixed oxides derived from hydrotalcite-like compounds (HTLCs) as catalysts received much attention in view of their unique properties [32]. HTLCs, also known as layered double hydroxides (LDHs), are a family of anionic clays. The chemical composition can be represented by the following general formula: [M(II)_1−*x*_M(III)_*x*_(OH)_2_]^*x*+^(A^*n*−^)_*x*/*n*_·mH_2_O, where M(II) and M(III) are divalent and trivalent cations in the octahedral positions within the hydroxide layers, *x* is the molar ratio M(III)/M_total_, and its value ranges between 0.17 and 0.33. A^*n*−^ is an exchangeable interlayer anion [33, 34]. HTLCs calcined at high temperatures will lose crystal water, and the interlayer anions and hydroxyl will be removed too. Therefore, the hydrotalcite-like layered structures will be destroyed, the surface area will increase, and the metal oxides are obtained. 

**Figure 1:**
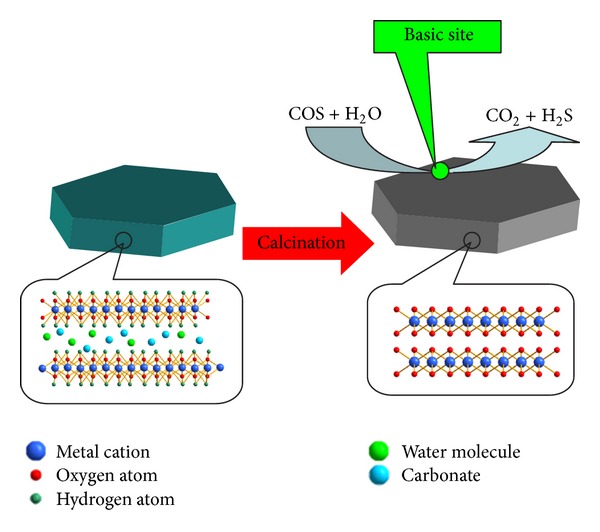
Hydrolysis of COS on the hydrotalcite-derived oxides.

In our previous studies, we found that the mixed oxide derived from HTLCs is a kind of potential catalysts for the hydrolysis of COS at low temperature (Figure 1). The catalyst performance was strongly related to the synthesis of pH and calcination temperature. In general, COS was hydrolyzed to H_2_S, which is depending on the chemistry of the derived oxides. The end products of hydrolysis were simple substance S and sulfate. The presence of oxygen will accelerate the formation of the final product [35–43].

#### 2.1.2. Activated Carbon

Activated carbon has become to be an ideal catalyst carrier because of its high specific surface area, the large pore volume, the large variety of surface functional groups, and good electron conductivity. However, the activity to remove COS for unmodified activated carbon is very low and its sulfur capacity is small; therefore, it must be modified. Activated carbon has not only excellent COS conversion rate, but also can remove H_2_S effectevily by using carbonates and other active ingredients to modify [44]. The 3018 desulfurizer developed by Dalian Institute of Chemical Physics showed a better removal COS performance at a temperature of about 80°C, the product was elemental sulfur [45, 46]. 

In our previous study, a series of microwave coal-based active carbon catalysts loaded by metal oxides were prepared by a sol-gel method and tested for the catalytic hydrolysis of COS at relatively low temperatures. The influences of preparation conditions on catalytic activity were studied, which were the kinds and amount of additive, calcination temperatures, and types and content of alkali [47–51].

#### 2.1.3. Special Carrier

Yan et al. have used honeycomb cordierite as the carrier in the experiment [52]. The cordierite was polished to form the pillar-shaped carrier, the desulfurizer which was made by using the method of dipping to make Al_2_O_3_ slurry load cordierite exhibit good desulfurization effect. The result indicated the following: COS hydrolysis activity gradually increases with the increase of the loading of *γ*-Al_2_O_3_ and La(OH)_3_ at low temperatures; airspeed has greater impact on the activity of the catalyst; La-Al/honeycomb cordierite catalyst has good antioxidant properties; the presence of O_2_ made the single loaded *γ*-Al_2_O_3_ catalyst lose some activation, while the catalyst which was combined with La-Al has not been affected; it indicated that La(OH)_3_ is COS hydrolysis catalyst which has higher antioxidant.

### 2.2. Active Ingredients

#### 2.2.1. Alkali Metal and Alkaline Earth Metal

Alkali metal and alkaline earth metal which are supported on *γ*-Al_2_O_3_ can modulate the distribution of the amount of alkali and base intensity, and they played slightly different roles: the alkali metal can modulate the amount of alkali apparently, while the alkaline earth metal can modulate the distribution of base intensity apparently.

George et al. found that a small amount of NaOH plays a significant role in promoting the COS hydrolysis; the initial rate of Co-Mo/Al_2_O_3_ catalyst which was impregnated by 3.9% NaOH can increase by 25 times at 230°C [23]. Li and Tan et al., and so forth, prepared a series of catalysts by using oxides which were impregnated with alkali metal and alkaline earth metal, and they sorted the catalytic activity by doing experiments: Cs_2_O/*γ*-Al_2_O_3_ > K_2_O/*γ*-Al_2_O_3_, BaO/*γ*-Al_2_O_3_ > Na_2_O/*γ*-Al_2_O_3_, and CaO/*γ*-Al_2_O_3_ > MgO/*γ*-Al_2_O_3_. The catalytic activity was also related to the amount of loaded metal oxides, and the activity reached the highest when the mole fraction is about 5% [53, 54].

#### 2.2.2. Transition Metals, Composite Metal Oxides, and Nanometals

Tong associated the activity of Al_2_O_3_ catalyst (a transition metal) with the position of transition metal promoter M in the periodic table, found the relationship of catalyst activity and the combination ability of the M–S bond, and concluded that Iron as the active component of the catalyst on the COS has the highest catalytic activity [55]. West et al. loaded a series of metal ions (Fe^3+^, Co^2+^, Ni^2+^, Cu^2+^, and Zn^2+^) to the Al_2_O_3_ plate. It was shown that Cu^2+^ has the catalyst activity only at the beginning of the reaction [56, 57]. In addition, researchers found that loading a variety of active components to the unified carrier can achieve better results. Wang et al. investigated a ferromanganese composite metal oxide by the coprecipitation method. It was shown that this desulfurizer has high-precision removal of COS and larger sulfur capacity in a strong reducing atmosphere. They also found that the desulfurization accuracy of the catalyst is greatly improved, while the nickel oxide and cerium oxide are added. The COS concentration of the outlet was less than 0.1 × 10^−6^; adding zinc oxide achieved a greater improvement of sulfur capacity [58]. With the development of nanotechnology, nanomaterials has began to be applied to the development of COS hydrolysis catalyst. Gao et al. investigated catalyst with *α*-FeOOH nanoparticles as the active ingredient, which was prepared by homogeneous precipitation method, the ammonia titration. They found that it has a good effect on the COS hydrolysis and a high activity under the condition of low temperature and high space velocity. Two series of catalysts achieved the 100% conversion of COS at the temperature of 60°C and 40°C–45°C, respectively, [59, 60].

#### 2.2.3. Rare Earth Metal Oxides

Colin Rhodesa et al. studied the synergistic effects of rare earth elements and alkaline rare earth accelerator on the Al_2_O_3_ plate. Through the analysis of DRIFT spectrum, it was shown that the role of hydroxyl groups on the surface composite catalyst is similar to pure Al_2_O_3_, and it was concluded that accelerator can provide high stable cation to the catalyst [61]. Zhang et al. initially investigated the hydrolytic activity of the rare earth series of sulfur oxides and divided the rare earth oxides into three categories, according to hydrolytic activity of COS: (1) high hydrolytic activity: La, Pr, Nd, and Sm; (2) high hydrolytic activity: Eu; (3) low hydrolytic activity: Ce, Gd, Dy, Ho, and Er, with the following order: La *≈* Pr *≈* Nd *≈* Sm > Eu > Ce > Gd *≈* Ho > Dy > Er. In addition, rare earth sulfur oxides showed a good oxidation resistance. Some researchers found that increasing temperature is conducive to improve the antioxidant capacity of the catalyst, but a certain amount of SO_2_ will lead to a decrease in catalyst activity, and it is a reversible inactivation [62–64].

## 3. Reaction Kinetics of COS Hydrolysis

Researches on catalytic hydrolysis reaction kinetics of carbonyl sulfide include establishing the kinetic equations of chemical reactions and determining the controlling step of hydrolysis. Dynamic models reported in articles are different because of the difference in research conditions. 

At present, most researchers hold that the catalytic hydrolysis of COS is a first-order reaction with respect to carbonyl sulfide, while the reaction order of water changes as the reaction conditions change. The controlling steps are also different because the reaction conditions such as concentration of carbonyl sulfide, reaction temperature, water-air ratio, and reaction atmosphere are different. 

Fiedorow et al. [65] studied the hydrolysis of carbonyl sulfide on pure alumina and base modified alumina, respectively. They concluded that the catalytic hydrolysis of carbonyl sulfide is a first-order reaction with respect to carbonyl sulfide and a zero-order reaction with respect to water. George [66] also reached the same conclusion by studying the hydrolysis of carbonyl sulfide on Co-Mo-Al catalyst. 

Tong et al. [67] studied the hydrolysis of carbonyl sulfide under medium temperature conditions and concluded that the catalytic hydrolysis of carbonyl sulfide is a first-order reaction with respect to carbonyl sulfide, while the reaction order of water is influenced by the partial pressure of water. When the partial pressure of water is 0.1–0.26, the reaction order of water is 0.4. When the pressure is higher than 0.26, the reaction order of water is −0.6. The controlling step under low temperature is the adsorption of carbonyl sulfide or the formation of reaction intermediated by adsorbed carbonyl sulfide and water. Chan and Dalld [68] studied the hydrolysis of carbonyl sulfide on Kaiser Kas201 catalyst. They adopted the single factor experiment method and drawn the same conclusion as Miroslav et al. [69] who studied the hydrolysis reaction kinetics of carbonyl sulfide on Co-Mo-Al catalyst and Al_2_O_3_ catalyst. They concluded that when the reaction temperature is 150 centigrade and when the volume fraction of carbonyl sulfide, hydrogen sulfides and carbon dioxide are 0.22%, 0.1%, and 0.01%, respectively, the hydrolysis rate remains unchanged as the fraction of water vapour increases from 0.2% to 2.5%. Thus, the reaction order of water is zero. 

Liang et al. [70] studied the hydrolysis of carbonyl sulfide on TGH-2 catalyst and found that when the water-air ratio is greater than 80, the reaction order of water is negative. Their explanation is that the micropore is blocked due to its condensation, so the reaction process cannot continue. Lin et al. [71] studied the hydrolysis of carbonyl sulfide under the conditions of low reaction temperature, high water-air ratio, and low carbonyl sulfide concentration. They concluded that the reaction order of carbonyl sulfide is 1, while the reaction order of water is −0.5. In other words, when the concentration of carbonyl sulfide is low, it will impede the hydrolysis process. Their explanations are that the condensation of water vapor blocks the pore of the catalyst and the two reaction sites are competitive, in which the adsorption of water on the basic site results in the decrease of catalytic activity. They held that the adsorption of carbonyl sulfide is dissociative and the adsorption isotherm corresponds with the Freundlich equation. In other words, the adsorption of carbonyl sulfide is the controlling step of the reaction.

Guo et al. [72] studied the hydrolysis of carbonyl sulfide on TGH-3Q catalyst and measured adsorption isotherm of carbonyl sulfide and water under the temperature of 20–70 centigrade. They concluded that the adsorption of carbonyl sulfide is dissociative and the adsorption isotherm corresponds with the Freundlich equation. The adsorption isotherm of water also corresponds with the Freundlich equation. However, the adsorption of carbonyl sulfide increases as the temperature increases, while the adsorption of water decreases as the temperature increases. The intrinsic kinetic equation of the hydrolysis is as follows:

(1) $$\begin{array}{c} R=kC_{\text{COS}}^{1}C_{{\text{H}}_{2}\text{O}}^{-0.5}\times \frac{1}{1+KC_{\text{C}{\text{O}}_{2}}}\\ k=1.82\;\times \;10^{14}e^{-75800/RT}\\ K=486e^{6000/RT}. \end{array}$$

In the equation, *R* means reaction rate; *k* means constant of reaction rate; *K* means adsorption constant of carbon dioxide; *C* means concentration of reactants.

The equation shows that the catalytic hydrolysis of carbonyl sulfide is a first-order reaction with respect to carbonyl sulfide. And the water is essential as the hydrolytic agent; however, excessive water will impede the reaction process. Besides, the hydrolysis speeds up as the temperature increases. Finally, the hydrolysis of carbonyl sulfide is controlled by surface adsorption.

By adopting the inner-recycle nongradient reactor, Liang et al. [70] studied the catalytic hydrolysis of carbonyl sulfide and found that the possibility of carbonyl sulfide to be adsorbed increases due to the high cycle ratio. Therefore, they concluded that hydrolysis is determined by surface reaction. By adopting the inner-recycle non-gradient reactor under the reducing atmosphere, Li et al. [73] studied the catalytic hydrolysis of carbonyl sulfide on *γ*-906 catalyst and found that the reaction orders of carbonyl sulfide and water are 0.66 and 0.06, respectively. They held that hydrolysis is controlled by both the internal diffusion and the chemical reaction. 

Williams et al. [74] found that the reaction kinetics of carbonyl sulfide also corresponds with the Langmuir-Hinshelwood equation. They held that the reactants are adsorbed on the surface of the catalyst, and the reaction between adsorbed carbonyl sulfide and water is the controlling step of the hydrolysis. By doing significance analysis, Tong S found that the Eley-Rideal model is more significant to the result compared with the Langmuir-Hinshelwood model.

## 4. Reaction Mechanism of COS Hydrolysis

The main hydrolysis reaction mechanism is shown as follows:

(2) $$\begin{array}{c} \text{COS}+{\text{H}}_{2}\text{O}\to \text{C}{\text{O}}_{2}+{\text{H}}_{2}\text{S} \end{array}$$

At present, it is widely believed that the hydrolysis of carbonyl sulfide belongs to base-catalyzed reaction, and the force of the base site has an important effect on the hydrolysis of carbonyl sulfide.

**Figure 2:**
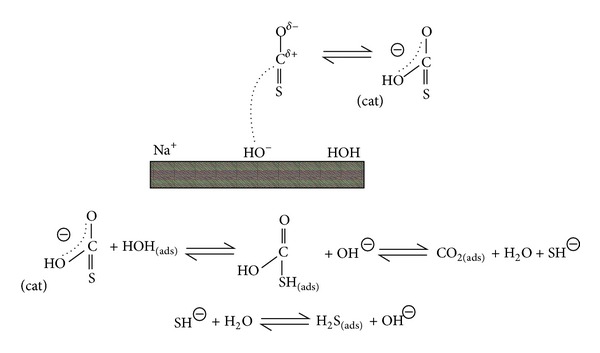
The concerted mechanism as proposed by George [23].

Williams et al. [74] have already studied the effect of surface alkalinity of alkali on the hydrolysis of carbonyl sulfide. They proposed the concerted mechanism to describe the hydrolysis of carbonyl sulfide (Figure 2). They held that hydroxyl and water exist on the surface of catalyst, and carbonyl sulfide is adsorbed due to ion-dipole interaction. The base site is the active site of carbonyl sulfide's hydrolysis. 

By adopting the IR spectrum method, Rhodes et al. [61] studied the hydrolysis of carbonyl sulfide on Co-Mo catalyst and found that the surface of the catalyst is covered partly by hydroxyl due to the adsorption of water, and the carbonyl sulfide is adsorbed due to ion-dipole interaction. Then, the Hydrogen Thiocarbonate (HTC) is formed. HTC is the intermediate product and will decompose into hydrogen sulfide and carbon dioxide. In the adsorption process, carbonyl sulfide and water are competitive. By adopting the insitu IR technology, Laperdrix et al. [75] studied the hydrolysis of carbonyl sulfide on Al_2_O_3_ catalyst and found that HTC is formed by the reaction of carbonyl sulfide and hydroxyl is adsorbed on the surface of the catalyst, which enhances the polarization of carbonyl sulfide.

By adopting the IR spectrum method, Fiedorow et al. [65] found that the hydroxyl on the surface of Al_2_O_3_ can adsorb carbonyl sulfide and the intermediate product is thiocarbonate which will decompose rapidly into hydrogen sulfide and carbon dioxide. Hydrogen sulfide and carbon dioxide are competitive in the adsorption process. Furthermore, they discovered that the activity of the catalyst decreases in acidic conditions, while it increases in alkaline conditions. They proposed the following reaction mechanism:

(3) $$\begin{array}{c} {\text{H}}_{2}\text{O}+\Theta ⟷{\text{H}}_{2}\text{O}\cdot \Theta \\ {\text{H}}_{2}\text{O}\cdot \Theta +\text{COS}\left(\text{g}\right)⟷{Η}_{2}\text{S}\cdot \Theta +\text{C}{\text{O}}_{2}\left(\text{g}\right)\\ {Η}_{2}\text{S}\cdot \Theta ⟷{Η}_{2}\text{S}+\Theta \\ \Theta :\text{active}\;\text{site}. \end{array}$$

Wang [76] studied the hydrolysis of carbonyl sulfide on mixed metal oxides and proposed the following hydrolysis mechanism of carbonyl sulfide:

(4) $$\begin{array}{c} \text{COS}+\Theta =\text{COS}\cdot \Theta \\ {\text{H}}_{2}\text{O}+\Theta ={\text{H}}_{2}\text{O}\cdot \Theta \\ {\text{H}}_{2}\text{O}\cdot \Theta +\text{COS}\cdot \Theta ={\text{H}}_{2}\text{S}\cdot \Theta +\text{C}{\text{O}}_{2}\cdot \Theta \\ {\text{H}}_{2}\text{S}\cdot \Theta ={\text{H}}_{2}\text{S}+\Theta \\ \text{C}{\text{O}}_{2}\cdot \Theta =\text{C}{\text{O}}_{2}+\Theta \\ \Theta :\text{active}\;\text{site}. \end{array}$$

Additionally, researchers found that the base strength on the surface of the catalyst has an important effect on the hydrolysis of carbonyl sulfide. By adopting the FTIR spectrum and quantum chemistry methods, Hoggan et al. [77] studied the hydrolysis of carbonyl sulfide on Al_2_O_3_ catalyst and found that hydrolysis proceeds much easier on sodium meta aluminate, which does not have bronsted acid. They concluded that carbonyl sulfide is mainly adsorbed on the site of weak base. Li et al. [78] studied the hydrolysis of carbonyl sulfide on alkali-modified *γ*-Al_2_O_3_ catalyst and found that distribution of base strength on the surface of the catalyst is related to the distribution of energy on the surface. They concluded that the effective range of base strength in the hydrolysis is between 4.8 and 9.8.

By adopting the CO_2_-TPD, Shangguan and Guo [79] studied the properties of the alkaline site on three kinds of Al_2_O_3_ catalyst and studied the hydrolysis of carbonyl sulfide and carbon disulfide. They found that the kinds, amounts, and strength of alkaline sites are different. The alkalescent site is the active site of the carbonyl sulfide's hydrolysis. The amount and strength of alkalescent site can be increased by supporting Pt and K_2_O on the surface of the catalyst; thus, the hydrolysis activity of the catalyst can be increased.

**Figure 3:**
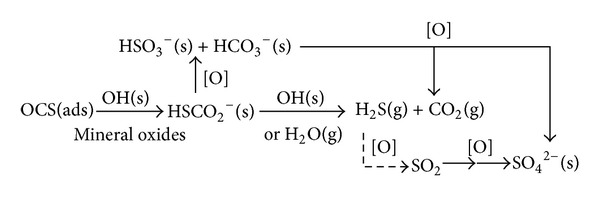
The concerted mechanism as proposed by He et al. [9, 24–27].

Hong He's group conducted in-depth studies on the reaction mechanism of the COS hydrolysis and they have achieved good results. By studying the oxygen toxicity mechanism of catalyst, they found that the hydroxyl on the surface of the catalyst plays an important role in the hydrolysis and the intermediate product is thiocarbonate. They reported that the reaction mechanism of COS on mineral oxides can be summarized as shown in Figure 3. 

In Figure 3, gaseous carbon dioxide (CO_2_), hydrogen sulfide (H_2_S), sulfur dioxide (SO_2_), surface sulfite (SO_3_^2−^), and sulfate (SO_4_^2−^) were found to be the gaseous and surface products, respectively. Hydrogen thiocarbonate (HSCO_2_^−^, HTC) was proposed to be the crucial intermediate for both the oxidation and the hydrolysis pathway [24–27, 80].

## 5. Conclusions

At present, the catalytic hydrolysis method is the main method to remove carbonyl sulfide. It has many advantages, such as low energy consumption, easy operation and less side reaction. The main task is to develop a new kind of catalyst which is highly active, highly stable, and highly antipoisoning. The catalyst carrier basically has two kinds. One is a delegate with Al_2_O_3_ and TiO_2_ metal oxides. Another kind of nonmetallic oxide is a delegate with activated carbon. Alkali metal and alkaline earth metal which are supported on *γ*-Al_2_O_3_ can modulate the distribution of the amount of alkali and base intensity. Regarding the production of catalytic hydrolysis of COS, the simple substance S and sulfate can be formed on the catalyst's surface, and the activity of the catalyst decreased when the S/SO_4_^2−^ species accumulated on the catalyst's surface. With the introduction of stringent requirements to reduce the sulfur content in industrial feed gas, the fresh impetus is being given to modifying and improving the existing preparation method of the hydrolysis catalyst. By studying the reaction kinetics and reaction mechanism, researchers can find better ways to develop the catalyst and to establish the desulfurization process. Therefore, the focus of reaction kinetics and reaction mechanism is to establish kinetic models under different conditions and to determine the effect of various kinds of factors and to clarify the path of the reaction.
